# Non-label immune cell state prediction using Raman spectroscopy

**DOI:** 10.1038/srep37562

**Published:** 2016-11-23

**Authors:** Taro Ichimura, Liang-da Chiu, Katsumasa Fujita, Hiroaki Machiyama, Tomoyuki Yamaguchi, Tomonobu M. Watanabe, Hideaki Fujita

**Affiliations:** 1 Laboratory for Comprehensive Bioimaging, RIKEN QBiC, 6-2-3 Furuedai, Suita, Osaka, Japan; 2 Department of Applied Physics, Osaka University, 2-1 Yamadaoka, Suita, Osaka, Japan; 3 Department of Chemistry, the University of Tokyo, 7-3-1 Hongo, Bunkyo-ku, Tokyo, Japan; 4 WPI, Immunology Frontier Research Center, Osaka University, 1-3 Yamadaoka, Suita, Osaka, Japan

## Abstract

The acquired immune system, mainly composed of T and B lymphocytes, plays a key role in protecting the host from infection. It is important and technically challenging to identify cell types and their activation status in living and intact immune cells, without staining or killing the cells. Using Raman spectroscopy, we succeeded in discriminating between living T cells and B cells, and visualized the activation status of living T cells without labeling. Although the Raman spectra of T cells and B cells were similar, they could be distinguished by discriminant analysis of the principal components. Raman spectra of activated T cells with anti-CD3 and anti-CD28 antibodies largely differed compared to that of naïve T cells, enabling the prediction of T cell activation status at a single cell level. Our analysis revealed that the spectra of individual T cells gradually change from the pattern of naïve T cells to that of activated T cells during the first 24 h of activation, indicating that changes in Raman spectra reflect slow changes rather than rapid changes in cell state during activation. Our results indicate that the Raman spectrum enables the detection of dynamic changes in individual cell state scattered in a heterogeneous population.

Fluorescence microscopy is an indispensable tool in biology and has greatly contributed to biological research for many years, as it is widely used for measuring cellular dynamics and status. Unless transgenic animals or cells bearing fluorescent proteins are used, labeling is required for fluorescence microscopy. Labeling is time-consuming and, in some cases, affects the natural behavior of the target. Thus, a cellular measurement tool with no need for fluorescent labeling would be highly preferred. Recently, Raman microscopy has attracted attention as a highly potent method for the label-free measurement of cells. Raman scattering originates from polarizability modulation due to intrinsic molecular vibrations. A Raman scattering spectrum is composed of peaks corresponding to molecular vibration modes with characteristic frequencies, from which one can deduce molecular species, composition, and concentration. The spectrum provides the intrinsic biochemical information of molecular compounds; therefore, there is no need for fluorescence labeling of target molecules. When implemented as a microscopy tool, Raman spectroscopy enables label-free, noninvasive analytical imaging of cells with single-cell sensitivity and resolution. In recent years, many groups, including ours, have reported the biological/biomedical applications of Raman scattering microscopy in cancer diagnosis^1^,^2^, cytochrome dynamics in apoptosis^3^, discrimination of normal and abnormal human sperm^4^, and in discrimination of cellular state upon differentiation^5^,^6^,^7^,^8^. Thus, Raman scattering microscopy has been revealed as an optional analytical tool in life sciences^9^. The advantage of Raman spectroscopy is now applied to immunology in this study.

Lymphocytes play a key role in acquired immunity to eradicate a wide variety of invasive pathogens. Among lymphocytes, CD4^+^ T cells recognize peptide-antigens presented on the major histocompatibility complex (MHC) with the T cell receptor (TCR) and produce cytokines to help or regulate immune cell activity, whereas B cells recognize soluble antigens with the B cell receptor and secrete specific antibodies against the antigen^10^. Activated CD8^+^ T cells produce cytotoxic molecules to kill the infected cells. For T cell activation, sufficient affinity of TCR with the peptide-MHC complex induces the assembly of TCR and CD3 molecules on the T cell membrane, resulting in tyrosine phosphorylation of CD3 and other associating molecules^11^. The TCR/CD3 signaling, together with co-stimulatory signaling via CD28 and/or cytokine signaling, initiates the expression of early activation markers, such as CD69, within several hours and results in cell-division within several days^12^. Activated T cells differentiate into several subpopulations characterized by the expression of particular cytokines, cytokine and chemokine receptors, and transcription factors to eradicate pathogens effectively^13^.

To understand this complex immune system, it is crucial to specify the cell type and state among heterogeneous populations at the single cell level in living and intact cells. However, conventional methods require staining of cells with specific antibodies or the extraction of RNA from bulk populations^14^. Surface marker staining is known to change the state of T cells even if the epitope for staining is carefully chosen. Therefore, a method that can predict cell state without staining is clearly desired. In this study, we have discriminated between T cells and B cells, and evaluated the extent of T cell activation by using Raman spectroscopy, which enabled the visualization of activated T cells without any labeling. The method described herein will be useful to determine the cellular response to stimuli without labeling.

## Results and Discussion

### Discrimination between T cells and B cells

**Figure 1:**
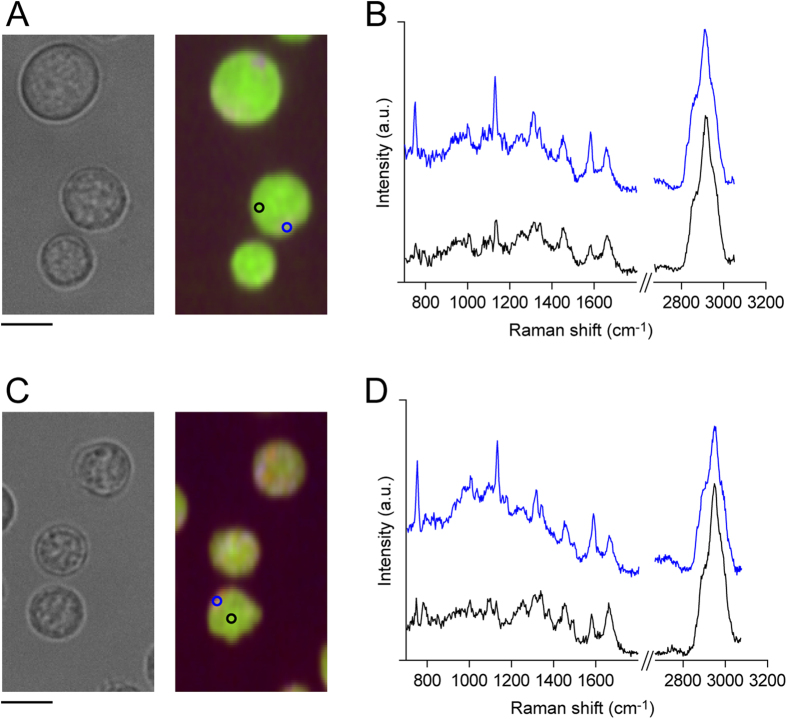
Raman images of T cells and B cells. (**A,C**) Bright field (left panel) and Raman images (right panel) of T cells (**A**) and B cells (**C**). Raman peaks at 753 cm^−1^ (cytochrome C), 2956 cm^−1^ (proteins), and 2852 (lipids) cm^−1^ are mapped in blue, green, and red, respectively. Scale bar, 5 μm. (**B,D**) Raman spectrum of a T cell (**B**) and B cell **(D**) obtained from the cytosolic region (blue) and the nuclear region (black). Raman spectra were obtained from the blue (cytosol) and black (nucleus) regions indicated in Raman images. The silent region (1800–2700 cm^−1^) is omitted from the graph.

**Figure 2:**
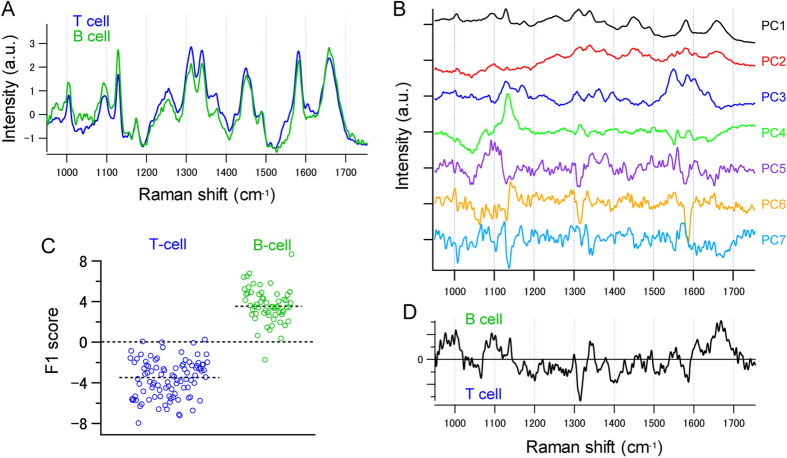
Raman spectrum from T cells and B cells. (**A**) Fingerprint region of the averaged Raman spectra obtained from 96 naïve T cells (blue) and 60 B cells (green). (**B**) The first 7 loading vectors calculated by PCA. (**C,D**) Result of DAPC of Raman spectra of naïve T and B cells. (**C**) Scatter plot of LDA scores for naïve T cells (blue) and B cells (green) along (**D**) the first discriminant axis (F1 vector). Each dot in (**C**) represents a single cell.

First, we compared the Raman spectra of T and B cells. Raman spectra were obtained using a home-built slit scanning microscope based on an inverted microscope (Ti Series, Nikon), equipped with a spectrometer (MK-300, Bunkou Keiki), as previously reported^3^. Figure 1A and C show Raman images of T and B cells, respectively, and Fig. 1B,D show representative Raman spectra from the cytosol and the nucleus. Although the Raman signal is stronger in the cytosolic region, in this study we averaged the spectra from the whole-cell region, and treated it as the Raman spectrum from a single cell. To compare the Raman spectra of T and B cells, Raman spectra from 96 T cells and 60 B cells from a DO11.10 mouse were averaged within cell-type, and were compared (Fig. 2A). The Raman spectra of these two cell-types were similar; however, differences were observed in some peaks, such as those at 1089, 1332, 1375, and 1483 cm^−1^.

To further investigate the differences in the Raman spectra between T and B cells, we performed a principal component analysis (PCA) against the fingerprint region of the Raman spectra, after background subtraction and standardization. All the Raman spectra were decomposed using principal component loading vectors (Fig. 2B). Reflecting the difference in Raman spectra between the T and B cells, T and B cells appear in different regions of the principal components (PC) score plots (Fig. S1).

To distinguish between T and B cells that showed similar spectra, we performed discriminant analysis of principal component (DAPC) on the obtained PCA scores. Figure 2C shows the distribution of the linear discriminant analysis (LDA) scores, and Fig. 2D shows the F1 vector along which the two classes (T and B cell) are best separated. The result of DAPC indicates that the two cell-types can be clearly separated. The error rate of the LDA was estimated to be 2.92%, through cross-validation with the leave-one-out method. Considering the fact that T and B cells are known to have heterogeneous populations with sub-fractions such as regulatory T cells or memory B cells, and that T and B cells, sorted without any fluorescence-probes contain some cell-type contamination, the discrimination of T and B cells using DAPC was successful.

### Raman spectrum of activated T cell

**Figure 3:**
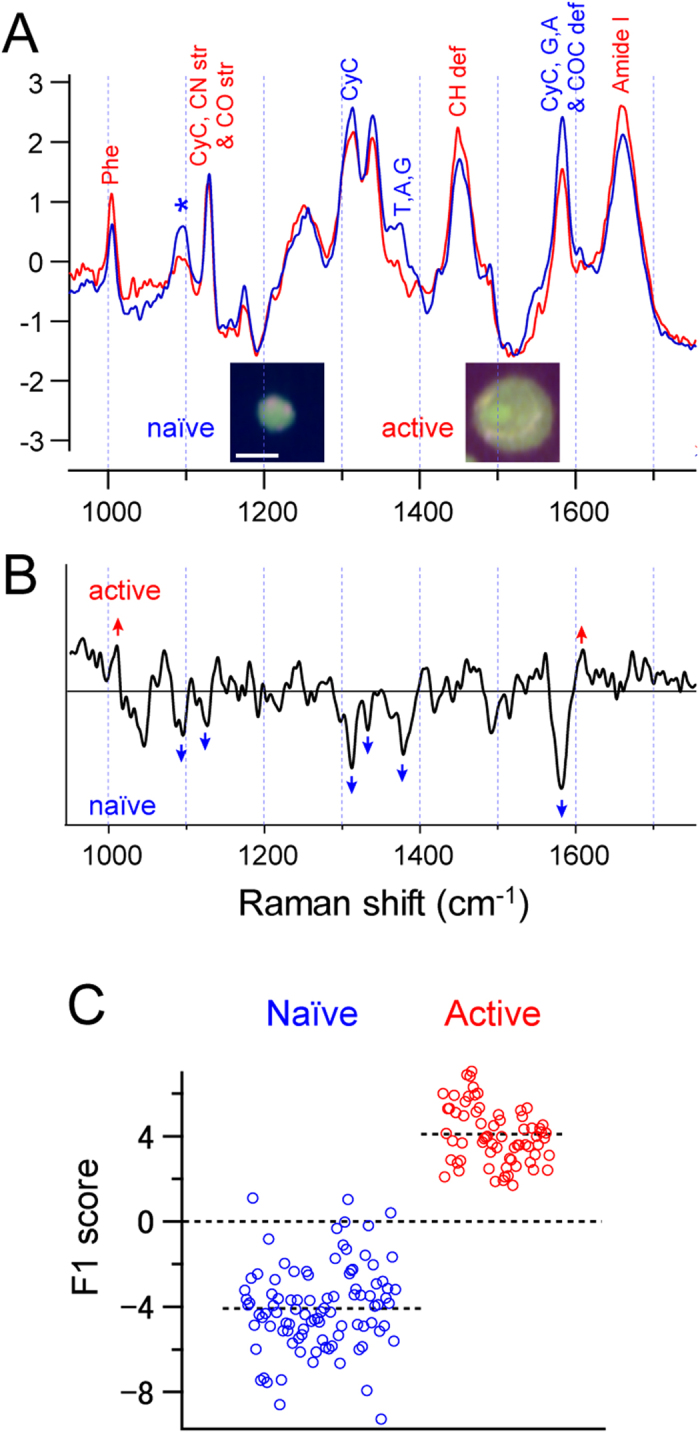
Raman spectrum of naïve and activated T cells. (**A**) Averaged Raman spectra of the fingerprint region of naïve (blue) and activated (red) T cells. Inset shows Raman images of naïve and activated T cells. Scale bar, 5 μm. (**B,C**) Result of DAPC of naïve and activated T cells. (**B**) First discriminant axis (F1 vector). (**C**) Scatter plot of LDA score along the first discriminant axis (F1 vector). Each dot in (**C**) represents a single cell.

We next assessed whether activated T cells could be discriminated from naïve T cells by Raman spectroscopy. We measured the Raman spectra of T cells from a DO11.10 mouse before and after activation with plate-bound anti-CD3 and anti-CD28 agonistic antibodies. DO11.10 mice express transgenic TCR on CD4^+^ T cells, which specifically reacts to a complex of ovalbumin antigen-peptide and MHC class II on the BALB/c background. Figure 3A shows the Raman spectra and Raman images of naïve CD4^+^ T cells, and those of T cells activated with anti-CD3 and anti-CD28 for 48 h. As represented in the Raman images, activated T cells were larger than the naïve T cells, which is a well-known activation signature. The Raman images also show the abundance of cytochrome c in activated T cells, indicating higher metabolic activity^15^. Therefore, Raman spectra differ between naïve and activated T cells (Fig. 3B).

The difference in Raman spectra between the naïve and activated T cells can be further visualized by a PCA, where the naïve and activated T cells appear at different locations in the score plots (Fig. S2). Using this PCA score, we performed DAPC, which resulted in a clear separation between the naïve and activated T cells along the direction of the F1 vector (Fig. 3C). The error rate of LDA was estimated to be 2.33%, indicating that activated T cells can be well distinguished by the Raman spectrum analysis. This result indicates that a difference in cell state can be visualized by Raman spectrum analysis.

### Visualizing the time course change in T cell activation

**Figure 4:**
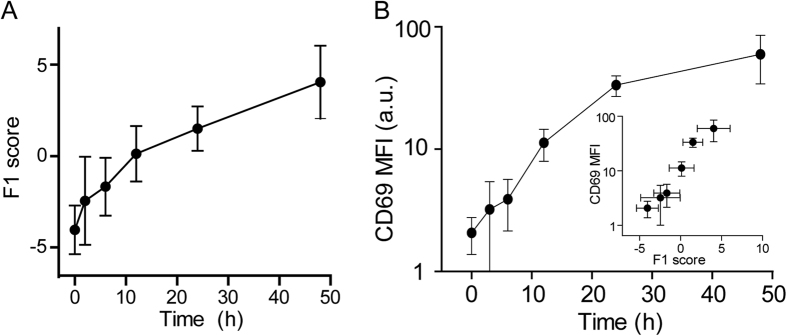
Time course change in the Raman spectrum during T cell activation. (**A**) Time course change in the LDA score during T cell activation. (**B**) Time course change in T cell activation assessed by CD69 expression. (Inset) Relationship between the LDA score and the T cell activation status as assessed by CD69 expression.

The ability to discriminate the intermediate state between naïve and activated T cells was evaluated by measuring the Raman spectra at different time-points during the activation of CD4^+^ T cells. The PC loading vectors calculated for the naïve T cells and the T cells activated for 48 h were used to construct the common state space. The Raman spectra of T cells activated for 2, 6, 12, or 24 h were then projected onto this state space by taking the inner product of each spectrum and the loading vectors. The inner product values that corresponded to the PCA scores were subjected to LDA. The calculated LDA score showed a gradual increase upon T cell activation (Fig. 4A). The LDA score change resembled the time course change in the logarithm of mean fluorescence intensity (MFI) of CD69 expression (Fig. 4B), which is an activation marker detected by flow cytometry using a specific antibody^16^. This result indicates that changes in Raman spectra reflect the global changes in cell state upon cellular activation at the population level, and can quantitatively represent the activation process.

**Figure 5:**
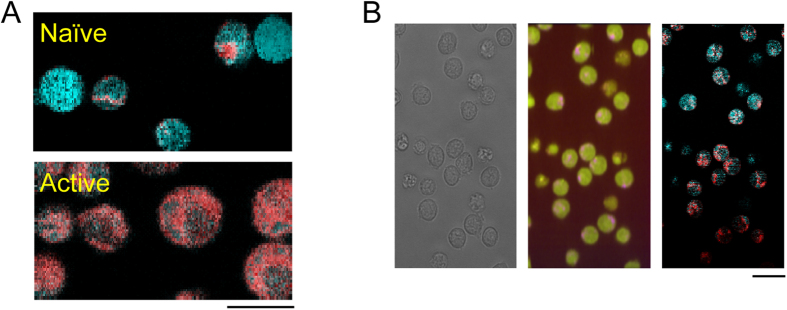
(**A**) Pseudo-colored image of naïve (upper) and 48-h activated T cells. Images are pseudo-colored such that the naïve state is represented in blue whereas the activated state is in red. (**B**) Bright field image (left), Raman image (middle), and activation status (right) image of T cells activated for 24 h. The Raman image is color-coded as in Fig. 1, and the activation image is color-coded as in (**A**).

As each pixel constituting the overall Raman image contains its own Raman spectrum, the above analysis can be also applied to each of these spectra. The logarithm of probabilities that the spectra belong to naïve or activated cells, which are defined in the manner of quadratic discriminant analysis (QDA), were linearly converted to blue-green and red channels of 8-bit RGB color images (the blue and green channels were synchronized), so that a cell closer to the naïve/activated state was colorized in blue/red. Figure 5A shows a representative pseudo-colored image of naïve and activated T cells. The activation status of T cells was clearly visualized by this method, making it easy to correctly identify the activated T cells. To investigate whether activated T cells could be distinguished in heterogeneous populations using this method, CD4^+^ T cells that were activated for a short period were imaged with the Raman microscope. Figure 5B shows T cells activated for 12 h visualized in the same way as in Fig. 5A. Although the T cells in this view look similar in the bright-field image and in the Raman image (except for some apoptotic cells), activated T cells can be distinguished in the pseudo-colored image. Thus, Raman microscopy enables not only the discernment of T and B cells, but also the monitoring of T cell activation in time; and space indicating that Raman microscopy has potential as an alternative method for fluorescent techniques such as flow cytometry.

## Conclusion

In this study, we demonstrated the use of Raman spectroscopy, in conjunction with DAPC to identify the activation status of living cells at the single-cell level. Once the state space is defined, cells of an unknown state can be validated in a non-invasive manner (*i.e.* without labeling). When reference spectra are carefully acquired, the present technique can be expanded into a range of disciplines in such a manner that it substitutes for immunofluorescence imaging.

## Materials and Methods

### Preparation of T cells and B cells

T cells and B cells were sorted from splenic cell suspensions from DO11.10 TCR transgenic mice on the BALB/c background with a typical purity of >90%. For CD4^+^ T cells, splenocytes were incubated with magnetic bead-conjugated antibodies against CD11b, CD11c, Ly6G, CD8, and B220 (Miltenyi Biotec, San Diego, CA), and the cells without magnetic beads were sorted using a MACS depletion-column (Miltenyi Biotec). Antibodies against CD11b, CD11c, Ly6G, CD8, and Thy1.2 were used for B cells. For T cell activation, CD4^+^ T cells were cultured on a plate pre-coated with antibodies against CD3 and CD28 (1 μg/ml each, from BD Biosciences, Franklin Lakes, NJ). All animal experiments were conducted according to the institutional guidelines for animal welfare as per protocols approved by the Animal Experiment Committee of Osaka University.

### Flow cytometry

T cells were stained with fluorescein (FITC)-conjugated anti-CD4 and allophycocyanin-conjugated anti-CD69 (1 μg/ml, from BD Biosciences) antibodies for 30 min. Propidium iodide (0.1 μg/ml, from Dojindo, Kumamoto, Japan) was added to remove the dead cells from the analysis before the detection of fluorescence intensity with the MACS Quant analyzer (Miltenyi Biotec). The data was analyzed and visualized using MACSQuantify software (Miltenyi Biotec).

### Raman imaging

Raman spectra were obtained with a home built slit scanning microscope based on a Nikon Ti microscope (Nikon, Tokyo, Japan) equipped with a spectrometer (MK-300; Bunkoh Keiki, Tokyo, Japan) as previously reported^3^. For observation, T cells and B cells were washed with Tyrode’s solution, transferred onto silica coverslips (SPI supplies, West Chester, PA), and observed through a 40× water immersion objective lens with 1.27 numerical aperture (Nikon CFP Plan Apo IR; Nikon). The sample was illuminated with a 532 nm laser at 2.4 mW/μm^2^. Laser power was carefully adjusted so that >90% of cells remain alive after observation. We further confirmed that cell state did not change significantly by laser illumination by comparing the Raman spectra of initial 5 line scans and last 5 line scans of naïve T cells. Typically, scanning of a single naïve T cell required 16~18 line scans, which took ~3 min. Figure S3 shows averaged Raman spectra (average of 40 cells) of initial and last 5 scans of naïve T cells, which were very similar (Fig. S3). Thus, it can be expected that cell state did not change significantly, at least when assessed by Raman spectra, during 3 min of the acquisition by laser exposure. The cells that died after observation were identified on a bright field image and were removed from the analysis.

### Data analysis

Throughout this study, we employed DAPC for spectral analysis, which utilizes the scores of PCA as explanatory variables of the discriminant analysis (DA). PCA plays the role of dimension reduction from raw spectral data to a set of PCA scores, and DA builds a state space for the discrimination of cells. Dimension reduction by PCA is essential to apply DA to multivariate data with a higher dimension than the number of samples, which is often the case with Raman spectroscopy of biological cells. The detailed procedure is described below. (1) The spectral analysis is started by subtracting background spectra from the measured raw spectra to extract the cellular Raman spectrum. In this study, all the background spectra mainly contain the Raman spectra from the silica glass substrate, ambient water, and the readout noise of the CCD detector used. For each Raman image, a mean Raman spectrum of 25 pixels far from the cell regions within the image was employed as a background spectrum, and was subtracted from the Raman spectra of all pixels in the image. (2) Raman spectra of the pixels in a cell region were averaged to represent the Raman spectrum of the cell. At this process, we judged the cell viability by the morphology observed in bright-field images, and only selected the viable cells for the next process. (3) Cellular Raman spectra were standardized by subtracting the mean value of each spectrum and dividing the spectrum by the standard deviation of the spectrum. (4) PCA was performed to decompose the cellular Raman spectra as a linear combination of PC loading vectors. We used the NIPALS algorithm to extract the PC loading vectors and the PC scores. (5) DA was performed for the PC scores to build a discrimination model. The LDA scores are given by the Fisher’s linear discriminant function, corresponding to the projection of the PC scores to the F1 axis.

For the spectral analysis of T and B cells, we used the processes from (1) to (5) and plotted the LDA scores of all spectra to evaluate the discrimination capability for both types of cells. On the other hand, for spectral analysis of the activated T cells, we collected Raman spectra at six stages of activation, including naïve cells, fully activated cells, and four intermediate states (2, 6, 12, and 24 hours after inducing activation). We used a dataset of the Raman spectra of the naïve and fully activated cells as the supervisor data, and built a PC space and a two-class DA space using the above processes (1)-(5), where the F1 axis represents the direction to best separate the two types of cells. For evaluating the discrimination capability for all six states, we further performed the following process. (6) The standardized Raman spectra at the four intermediate states were projected to the already-built PC space to obtain the PC scores by taking the inner product with the PC loading vectors. (7) The PC scores were then converted to F1 scores of the already-built DA space. The LDA scores for all six states were plotted as a function of time after the induction of activation.

For clear visualization of activation status, Raman images were reconstructed with pseudo-colors based on the above analysis. Each of the spectra at all pixels of the Raman images, including the pixels outside the cell region, was projected into the DA space by applying the above processes (6)-(7). The logarithm of probabilities that the spectrum belongs to naïve or activated cells were linearly converted to blue-green and red channels of 8-bit RGB color image (blue and green channels were synchronized) so that a cell closer to the naïve/activated state was depicted in blue/red.

## Additional Information

**How to cite this article**: Ichimura, T. *et al*. Non-label immune cell state prediction using Raman spectroscopy. *Sci. Rep.* **6**, 37562; doi: 10.1038/srep37562 (2016).

**Publisher's note:** Springer Nature remains neutral with regard to jurisdictional claims in published maps and institutional affiliations.

## Supplementary Material

Supplementary Figures
