# Supplementary material for: Non-label immune cell state prediction using Raman spectroscopy
Source: Sci Rep. 2016 Nov 23;6:37562. doi: 10.1038/srep37562 (PMC5120326; doi:10.1038/srep37562)
Supplement: Supplementary Figures [file srep37562-s1.doc]

**Supplementary Information:**

**Non-label immune cell state prediction using Raman spectroscopy**

Taro Ichimura^1^, Liang-da Chiu^2,3^, Katsumasa Fujita^2^, Hiroaki Machiyama^4^, Tomoyuki Yamaguchi^4^, Tomonobu M. Watanabe^1^, and Hideaki Fujita^1,4*^

^1^*Laboratory for Comprehensive Bioimaging, Riken QBiC, 6-2-3 Furuedai, Suita, Osaka, Japan*

^2^*Department of Applied Physics, Osaka University, 2-1 Yamadaoka, Suita, Osaka, Japan*

^3^Department of Chemistry, the University of Tokyo, 7-3-1 Hongo, Bunkyo-ku, Tokyo, Japan

^4^*WPI, Immunology Frontier Research Center, Osaka University, 1-3 Yamadaoka, Suita, Osaka, Japan*

Short title: Detecting T cell status using Raman spectroscopy

*Corresponding author: Laboratory for Comprehensive Bioimaging, Riken Quantitative Biology Center, OLABB, 6-2-3 Furuedai, Suita, Osaka 565-0874, Japan. Tel: +81-6-6155-0111. Fax: +81-6-6155-0112. E-mail: hideaki.fujita@riken.jp

**Key words: B cell; immune response; single cell analysis; Raman microscopy; discriminant analysis; principal component analysis**

**Figure S1.** Score plot of PCA result against naïve T cell (blue) and naïve B cell (green) Raman spectrum. Each dot show single cell.

**Figure S2.** Score plot of PCA result against naïve (blue) and activated (red) T cell Raman spectrum. Each dot show single cell.

**Figure S3.** Averaged Raman spectra (average of 40 cells) of initial 5 line scans (blue) and last 5 line scans (red) of naïve T cells. Typically, scanning of a single naïve T cell required 16~18 line scans, which took ~3 min.
